# A number-form area in the blind

**DOI:** 10.1038/ncomms7026

**Published:** 2015-01-23

**Authors:** Sami Abboud, Shachar Maidenbaum, Stanislas Dehaene, Amir Amedi

**Affiliations:** 1 Department of Medical Neurobiology, The Institute for Medical Research Israel-Canada, Faculty of Medicine, The Hebrew University of Jerusalem, Jerusalem 91220, Israel; 2 Collège de France, 11 Place Marcelin Berthelot, 75005 Paris, France; 3 Institut National de la Santé et de la Recherche Médicale, Cognitive Neuroimaging Unit, 91191 Gif sur Yvette, France; 4 Commissariat à l’Energie Atomique, Division of Life Sciences, Institute of Bioimaging, Neurospin, 91191 Gif sur Yvette, France; 5 Université Paris 11, 91401 Orsay, France; 6 The Edmond and Lily Safra Center for Brain Sciences (ELSC), The Hebrew University of Jerusalem, Jerusalem 91220, Israel; 7 The Cognitive Science Program, The Hebrew University of Jerusalem, Jerusalem 91220, Israel; 8 Sorbonne Universités, UPMC Univ Paris 06, Institut de la Vision, UMR_S 968, Paris F-75012, France

## Abstract

Distinct preference for visual number symbols was recently discovered in the human right inferior temporal gyrus (rITG). It remains unclear how this preference emerges, what is the contribution of shape biases to its formation and whether visual processing underlies it. Here we use congenital blindness as a model for brain development without visual experience. During fMRI, we present blind subjects with shapes encoded using a novel visual-to-music sensory-substitution device (The EyeMusic). Greater activation is observed in the rITG when subjects process symbols as numbers compared with control tasks on the same symbols. Using resting-state fMRI in the blind and sighted, we further show that the areas with preference for numerals and letters exhibit distinct patterns of functional connectivity with quantity and language-processing areas, respectively. Our findings suggest that specificity in the ventral ‘visual’ stream can emerge independently of sensory modality and visual experience, under the influence of distinct connectivity patterns.

## Abstract (web summary)

The human visual cortex includes areas with preference for various object categories. Here, Abboud *et al*. demonstrate using visual-to-music substitution, that the congenitally blind show a similar preference for numerals in the right inferior temporal cortex as sighted individuals, despite having no visual experience.

## Graphical abstract

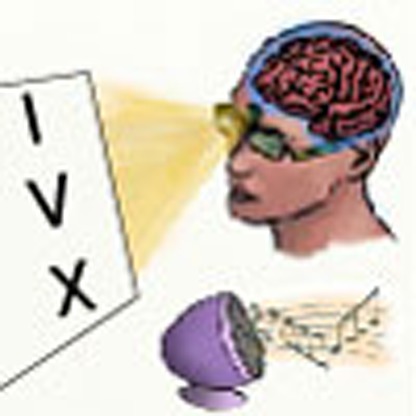

The ventral stream of the visual cortex includes areas with strong preference for various visual categories of objects^1^,^2^,^3^,^4^,^5^,^6^,^7^. Most notably, this preference was shown for faces in the fusiform face area^5^,^8^ (FFA) and occipital face area^9^, for body parts in the extrastriate body area^2^ and for letter strings in the visual word-form area^1^ (VWFA). An intriguing addition to this body of research is the recently reported preference for visual numerals in the right inferior temporal gyrus (rITG), a region therefore labelled as the visual number-form area (VNFA). Using electrocorticography (ECoG), it was demonstrated that this area has a significantly higher response to Arabic numerals when compared with letters or false fonts, and when compared with words with a similar semantic or phonetic content^10^. The existence of this area was predicted in a study of a subject with a number-reading deficit^11^ and later by the triple-code model for number processing^12^. It was argued that this area was not detected using fMRI due to signal dropout in the inferior temporal cortex^10^, although activation at or near this site can in fact be seen, in retrospect, in a contrast of Arabic versus verbal numerals^13^ and in large-scale databases of calculation fMRI^14^. A topographical dissociation between letter and digit recognition is also congruent with the occasional sparing of numerals in pure alexic patients^15^,^16^,^17^, and with the distinct hemispheric specialization in early visual processing for each of these categories^18^.

The aforementioned studies assigned the apparent cortical preference to specialized *visual* subprocesses. The VWFA, for example, was hypothesized to be sensitive to the visual characteristics of scripts such as line junctions^19^, foveal position^20^ and high spatial frequencies^21^.

However, recent studies by our group and by other laboratories have shown that the cortical preference in the ‘visual’ cortex might not be exclusively visual and in fact might develop independently of visual experience^22^,^23^,^24^,^25^,^26^. Specifically, an area showing preference for reading, at the precise location of the VWFA, was shown to be active in congenitally blind subjects during Braille reading^27^ and during reading using auditory sensory-substitution first learned in adulthood^28^. Other organizational principles such as the division of labour between the dorsal and ventral streams and the large-scale segregation of the ventral stream into animate and inanimate semantic categories have also been shown to be independent of visual experience^29^,^30^. More generally, an overlap in the neural correlates of equivalent tasks has been repeatedly shown between the blind and sighted using different sensory modalities^31^,^32^,^33^,^34^,^35^,^36^,^37^,^38^.

These findings lead us to hypothesize that the development of a cortical preference for number symbols may not rely on a computation that is visual in nature. Our hypothesis is that the VNFA deciphers symbols for the purpose of connecting them to a quantity representation. Therefore, such a specialization may exist for all shapes that act as numbers, including for instance Roman numerals (letters that act as numbers), and even if these shapes are not conveyed through the visual modality.

Identifying a systematic preference for number symbols, distinct from the preference for letters, which would persist in the congenitally blind at the same location as in the sighted, would undermine theories that rely on visual input as a determining factor for developmental preferences in the ventral visual cortex. Considering that numerals and letters have very similar shapes, which are only attached to different domains by an arbitrary cultural convention, we propose that the explanation for having two distinct areas, the VNFA with preference for numerals and the VNFA with preference for letters, may lie in the functional circuit that each area is connected with: the VNFA would preferentially connect to a quantity-processing network, previously localized to the intraparietal sulcus^39^,^40^, whereas the VWFA would preferentially connect to a language-processing network.

To test these predictions, we recruited nine blind subjects (see blindness characteristics in Supplementary Table 1) and trained them on the EyeMusic sensory-substitution device (SSD, see Methods). We developed this novel SSD^41^ to enable the encoding of the spatial geometrical features of images using time and pitch, that is, musical note, and their colour using various timbres, that is, different musical instruments (Fig. 1a). We asked subjects to attend to identical stimuli, while performing three distinct *Numeral, Letter* and *Colour* identification tasks during fMRI blood-oxygen-level-dependent (BOLD) signal acquisition (Fig. 1b). We specifically used physically identical stimuli during all tasks to reveal task-specific activations and avoid possible biases due to the nature of the symbols used. Given the BOLD signal dropout in the temporal areas of investigation, we developed a method for preprocessing the data that exclude voxels with large signal dropout at the single-subject level (see Methods). We then examined the preferential brain activation when performing each task in comparison with the others. Furthermore, we acquired resting-state fMRI and analysed the functional connectivity of the VWFA and VNFA in the same group of blind subjects and a group of sighted subjects.

To anticipate on the results, we find greater activation in the rITG when the congenitally blind perform the *Numeral* task compared with the other tasks, at a location similar to the rITG site showing numeral selectivity in the sighted^10^. Hence, we show that visual experience is not necessary to observe a specialization for number symbols in the VNFA. We also show that the VNFA is specifically connected with the quantity-processing network and the VWFA is specifically connected with the language-processing network in both congenitally blind and sighted individuals. This is in accordance with our proposal that the distinct specificity for numerals and letters emerges under the influence of connectivity patterns independently of sensory modality and visual experience.

## Results

### Training and task

**Figure 1:**
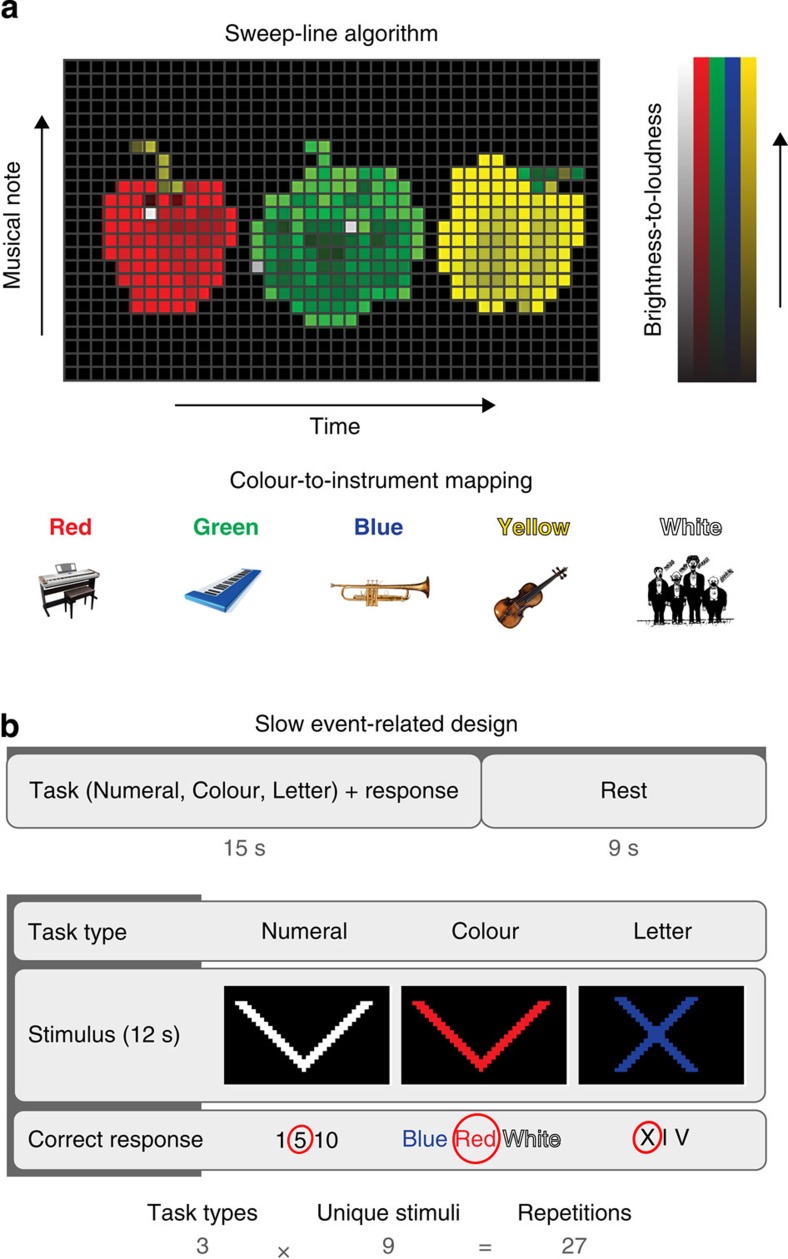
Methods. (**a**) The EyeMusic visual-to-music sensory substitution—*Sweep-line algorithm:* an input image is first resized to 40 × 24 pixels and then colour is clustered to the nearest of the colours listed below. Afterwards, the 24 rows are mapped on musical notes of corresponding instruments, which are played sequentially column-after-column. Pixel brightness is mapped to sound loudness, as demonstrated by the bars on the right side. In the middle, a sample image of three peppers resized and colour-clustered. *Colour-to-instrument mapping:* each of the colours is mapped to a musical instrument. Blue is mapped to brass instruments (Tuba, Trombone and Trumpet), yellow to string instruments (Cello and Violin), red to ‘Rapman’s Reed’, green to ‘Reggae Organ’, white to choir (Bass, Tenor, Alto and Soprano) and Black is mapped to Silence. (**b**) Experimental design—*top:* the slow event-related fMRI experiment design that consists of 27 trials of stimulation, response and rest periods. *Bottom:* task types, sample stimuli and the correct answer for each case according to the task type and stimulus. Identical stimuli were used in all tasks.

Each of the nine blind subjects had 25–30 h of basic training on the EyeMusic, during which they learned to hear coloured shapes, that is, to construct basic shapes and to identify their colours. Upon training completion, all blind subjects underwent an fMRI session that consisted of a resting-state acquisition and two sequences of slow event-related experiment acquisitions during which the subjects carried out three identification tasks. Crucially, the exact same stimuli were used under all tasks (Fig. 1b). The stimuli were the shapes I, V and X in three different colours. Subjects were instructed either to identify the *Colour* of the shape presented, or to interpret it as a *Latin letter* or as a *Roman numeral*, according to an auditory instruction announcing the trial type at the beginning of each trial. Each subject listened to the stimuli and had to choose Blue, Red or White in the *Colour* task, I, V or X in the *Letter* task and 1, 5 or 10 in the *Numeral* task (Fig. 1b; further detailed in Methods).

### Behavioural results

Performance levels in the *Numeral*, *Colour* and *Letter* tasks were 90±5%, 86±21% and 89±14%, respectively (mean±s.d.; see single-subject performance in Supplementary Table 2). Performance in all tasks was well above chance (Wilcoxon, *P*<0.0005) demonstrating the ability to perform each task correctly. None of the task pairs showed any statistical difference in performance levels (one-way analysis of variance (ANOVA); *P*=0.87).

### Slow event-related imaging experiment

**Figure 2:**
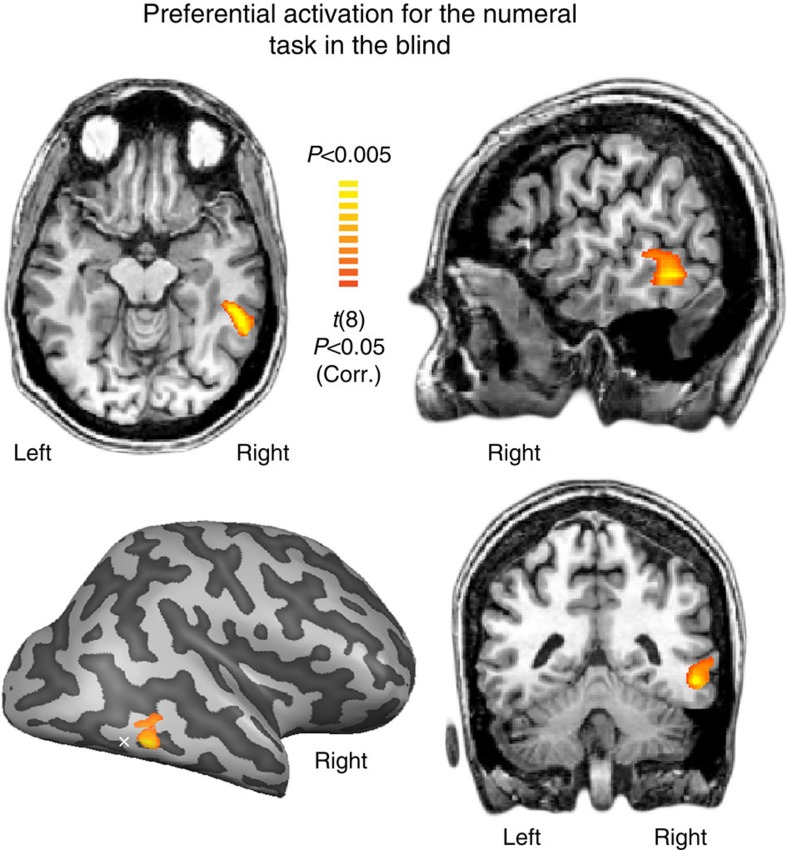
Preferential activation for the *Numeral* task in the blind. The result of the random-effects group analysis (corrected for multiple comparisons) in a group of blind subjects (*n*=9) for the *Numeral* task versus the *Colour* and *Letter* tasks contrast. The auditory stimuli I, V and X were encoded by the EyeMusic SDD and were identical in all the three tasks. Sagittal (*x*=53), coronal (*y*=−44) and transverse (*z*=−12) slices are shown, in addition to an inflated brain view of the lateral side of the right hemisphere. A single significant area with preferential activation for number symbols was found in the rITG (peak coordinates *x*=53, *y*=−44, *z*=−12; Table 1; Supplementary Table 3). The white cross indicates the location of the peak of the activated found in ref. 10.

Contrasting the *Numeral* task versus the *Colour* and *Letter* tasks using a general linear model (GLM) random-effects group analysis and correction for multiple comparisons across the entire cortex shows only one significantly activated area (Fig. 2, Table 1). This area in the rITG (peak coordinates *x*=53, *y*=−44, *z*=−12) shows larger activation for the numeral identification task (Size: 2,665 voxels, *t*=4.29, *P*<0.003; Fig. 2; Table 1; refer to Supplementary Fig. 1 for average percent BOLD signal change). The peak of this area is less than 4 voxels away from the VNFA identified in ref. 10 on the basis of its preference for visual stimuli of Arabic number symbols^10^ (peak coordinates *x*=50, *y*=−55, *z*=−13). Contrasting the *Numeral* task only versus the *Letter* task shows the same area in the rITG to be the only significantly activated area (Supplementary Table 3).

Inspecting the *Numeral* versus the *Colour* and *Letter* tasks contrast map without correction for multiple comparisons also revealed a symmetrical area in the left ITG (peak coordinates *x*=−56, *y*=−50, *z*=−8). In addition, also not surviving the correction for multiple comparisons, we observed a bilateral parietal preferential activation in the intraparietal sulcus (Supplementary Fig. 2; Supplementary Table 4).

A control analysis using a protocol that divides each event into three parts (instruction, stimulus and response; See Methods and Supplementary Fig. 3A) shows that it is not the mere instruction to think about numerals that induces the activation shown above but the actual processing of the task (Supplementary Fig. 3B).

Contrasting the *Letter* task versus the *Colour* and *Numeral* tasks using the usual random-effects group analysis did not yield any significant clusters. However, a fixed-effects group analysis demonstrated preferential activation in the VWFA within the occipitotemporal sulcus (which was the only activation in the visual cortex), in addition to clusters in the left inferior and middle frontal gyri and superior temporal gyrus (*P*<0.05 Bonferroni corrected; Supplementary Fig. 4; Supplementary Table 5).

### Functional connectivity analysis

**Figure 3:**
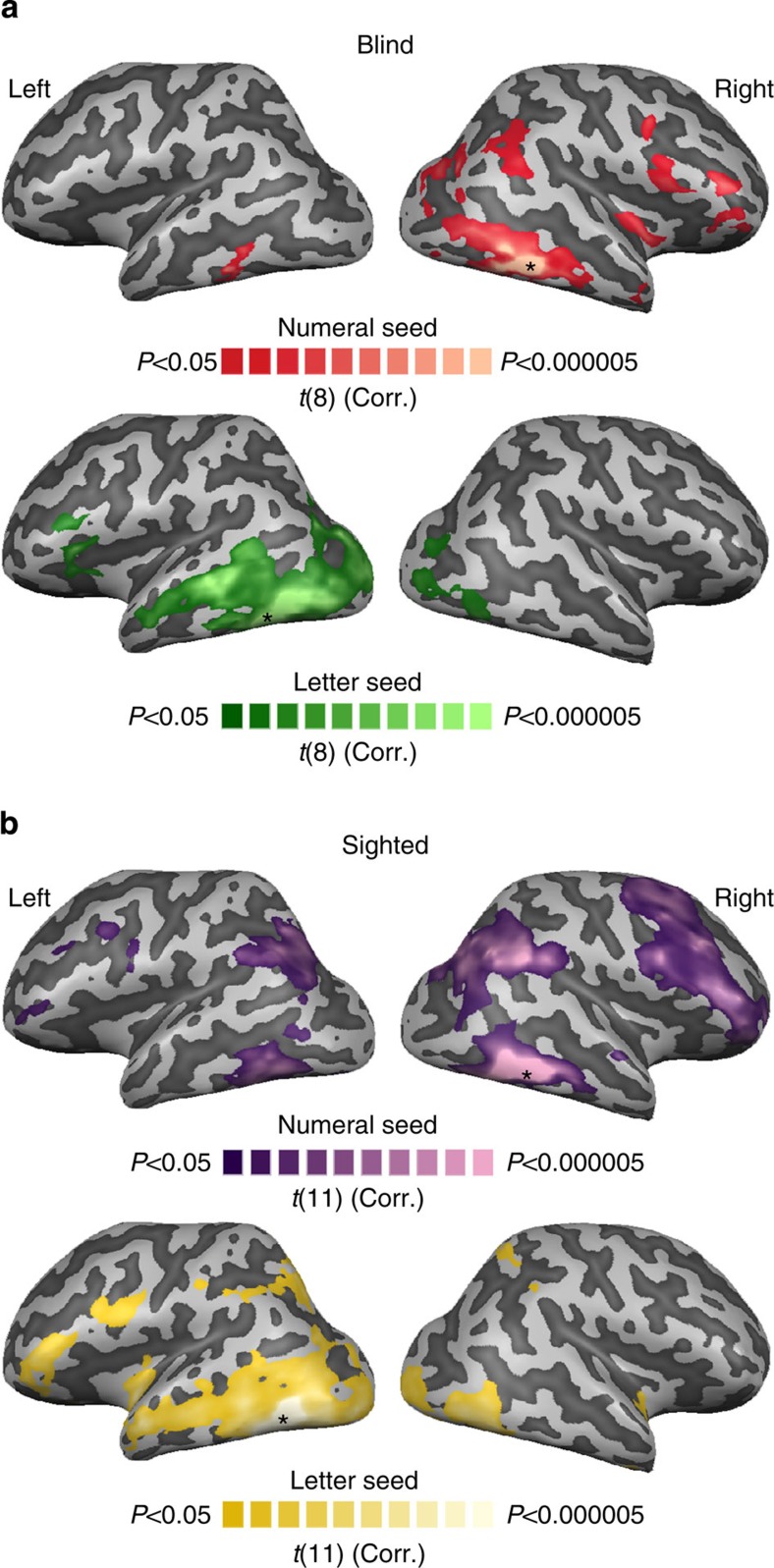
Functional connectivity results. The result of the random-effects group analysis (corrected for multiple comparisons) of functional connectivity to the seeds defined by (1) the peak of the area active for the *Numeral* task, denoted as the *Numeral seed* and (2) the peak of the area active for the *Letter* task, denoted as the *Letter seed*. A lateral view of an inflated brain overlaid with the connectivity maps and an asterisk marking the relevant seed. (**a**) *Blind group (n=9)*—functional connectivity map from the *Numeral seed* is shown in red, and from the *Letter seed* in green. (**b**) *Sighted group (n=12)*—functional connectivity map from the *Numeral seed* is shown in purple, and from the *Letter seed* in yellow. Both groups show a co-activation of the *Numeral seed* with areas involved in quantity-processing and the *Letter seed* with areas involved in language-processing.

Using a seed in the VNFA (*Numeral seed*; defined by the *Numeral* task contrast peak) and regressing out a seed in the letter-selective VWFA (*Letter seed*; defined by the *Letter* task contrast peak) to remove any shared correlations (partial correlation), shows specific co-activation of the VNFA with the right intraparietal sulcus (rIPS; an area involved in number processing) as well as to the homologue area in the lITG, large parts of the right temporal cortex, the right middle occipital gyrus, the right inferior frontal gyrus and sulcus and the right insula (Fig. 3a—Red; Supplementary Fig. 5A). The reciprocal functional connectivity analysis seeded from the VWFA, regressing out the correlation from the VNFA, showed co-activation with the left temporal cortex and the left inferior frontal gyrus (areas involved in language-processing) in addition to left occipital cortex and a cluster in the posterior right occipital cortex (Fig. 3a—Green; Supplementary Fig. 5B).

We also compared the functional connectivity from the two seeds and generated the cortical preference map that shows the significant differences between them. The results of the comparison show that the *Numeral seed* is significantly more co-activated with the rIPS when compared with the *Letter* seed and the *Letter seed* is more co-activated with the temporal and occipital cortices when compared with the *Numeral seed* (Supplementary Fig. 6A).

In order to determine whether this result is specific to the blind, we ran the same functional connectivity analysis, using the same seeds, on a group of sighted subjects. Similarly to the blind group, connectivity from the *Numeral seed* shows co-activation mainly with the bilateral parietal cortex (the intraparietal sulci), the homologue area in the lITG and the right frontal cortex (Fig. 3b—Purple; Supplementary Fig. 5C), while functional connectivity from *Letter seed* shows co-activation mainly with the left temporal and occipital cortices, left inferior frontal cortex and bilateral insula (Fig. 3b—Yellow; Supplementary Fig. 5D). The comparison of functional connectivity between the two seeds in the sighted shows similar results to the comparison in the blind. Specifically, the *Numeral seed* is significantly more co-activated with both the right and left IPS when compared with the *Letter* seed, and the *Letter seed* is more co-activated with the temporal and occipital cortices when compared with the *Numeral seed* (Supplementary Fig. 6B). Repeating the same analyses in the blind and sighted using the Pearson product–moment correlation coefficient instead of the partial correlation coefficient shows only minor differences in the functional connectivity pattern (Supplementary Figs 7 and 8).

To ensure that the distinct areas showing co-activation for the *Numeral* and *Letter* seeds do not arise because of the fact that the *Numeral* seed is in the right hemisphere and the *Letter* seed is in the left hemisphere, we ran the same analysis using symmetrical seeds. We defined the symmetrical seeds by changing the sign of the *x*-coordinate for each seed. The results show that the *Numeral seed* in the rITG and the *Sym. Letter seed* in the right occipitotemporal sulcus have different functional connectivity maps (Supplementary Fig. 9). Similarly, the results show that the Sym. *Numeral seed* in the left ITG and the *Letter seed* in the left occipitotemporal sulcus have different functional connectivity maps (Supplementary Fig. 9). This functional connectivity pattern, in the blind and the sighted, suggests that the distinction between the *Numeral seed* and the *Letter seed* maps is not due to a hemispheric bias.

## Discussion

We used fMRI to study the processing of number symbols and letters in the congenitally blind using visual-to-music sensory substitution. We explored the whole-brain activation when blind subjects were presented with coloured shapes, encoded by the EyeMusic SSD, and asked to perform distinct tasks on the very same stimuli (letters I, V and X): identifying the *Colour* of the shape, the quantity of the Roman *Numeral* it represents or the *Letter* it represents. When contrasting the *Numeral* task versus the other two tasks, subjects show a preferential activation in the rITG (Fig. 2, Table 1). The only activation across the entire brain for this contrast peaked in close vicinity to the site of preference for numerals previously identified in sighted individuals using ECoG and employing a completely different methodology^10^. This result demonstrates that visual experience is not necessary to observe a specialization for number symbols in the VNFA. Furthermore, it excludes an interpretation relying on confounds due to differences in the stimuli, because the preference was present despite the use of identical stimuli in all tasks.

These two points taken together suggest that neither the sensory-input modality and visual experience, nor the physical sensory stimulation itself, play a critical role in the specialization observed in this area.

Crucially, we hypothesized that what matters is the circuitry in which areas are engaged, which biases some ventral visual sites towards acquiring visual preferences. To test this idea, we used the peak of the activated ventral inferotemporal area for the numeral task and the peak of the activated occipitotemporal area for the letter task as seeds for a resting-state functional connectivity analysis in the group of congenitally blind, as well as in a group of sighted individuals. The results showed a co-activation of the *Numeral seed* with the rIPS in the parietal cortex (Fig. 3a,b), which has been repeatedly implicated with the representation of quantities^39^,^40^. The *Letter seed*, on the other hand, was co-activated with the left temporal cortex and the left inferior frontal gyrus (Fig. 3a,b) that have been shown to be involved in language processing^42^,^43^. Maps from both seeds show a striking resemblance between the congenitally blind and the sighted groups. Control analyses further showed that these distinct connectivity maps are neither due to a hemispheric bias that depends on the lateralization of the seeds (Supplementary Fig. 9) nor due to the use of partial correlation (Supplementary Figs 7 and 8—using the Pearson product–moment correlation coefficient).

In the following paragraphs, we will discuss the finding of a VNFA in the blind, and the implications of the functional connectivity between the VNFA to quantity-processing areas and the VWFA to language-processing areas on theories involving the emergence of separate neural circuits processing numerals and letters.

Using fMRI, we show a preferential activation for the task of identifying number symbols in the rITG, thus verifying the results of a recent paper showing this preference using ECoG^10^. Whereas the coverage of ECoG is limited to the clinically oriented placement of electrodes, fMRI provides a whole-brain coverage, which shows that the area in the rITG is not only preferentially active for number symbols but it is also the only area showing a significant preference (Fig. 2), surviving the conservative correction for multiple comparisons. The uncorrected results revealed bilateral activation in the IPS in addition to an area in the lITG that is homologous to the significant activation in the rITG (Supplementary Fig. 2; Supplementary Table 4).

Previous research using fMRI experienced difficulties in identifying the number-form area in rITG, presumably because this region is highly sensitive to magnetic susceptibility artifacts^10^. An fMRI experiment by Polk *et al*.^44^ utilized a single-subject approach when analysing a contrast between number strings versus consonant strings in a same/different judgment task. Their results were inconclusive, as activation occupied varying locations in the visual cortex in only three out of the five subjects that participated in the task^44^. Park *et al*.^45^, utilizing a similar same/different judgment task, showed a preference for number strings versus consonant strings in the right occipital cortex^45^ (peak coordinates *x*=49, *y*=−72, *z*=3). This location is superior and far more posterior to the preference we show in the rITG. However, calculation experiments not directly aimed at revealing a number-form computing area showed right ventral stream activation in vicinity to the location of our result. Pinel *et al*.^13^ contrasted Arabic versus verbal numerals in a numerical comparison task and reported a greater activation to Arabic numerals in the right fusiform gyrus^13^ (peak coordinates *x*=44, *y*=−64, *z*=−1), medial and slightly posterior to the preference we show in the rITG. In a different study of a subtraction calculation task^14^, a cluster was observed in the rITG (peak coordinates *x*=53, *y*=−58, *z*=−8) at a location slightly posterior to the preference we show in the rITG for the task of identifying number symbols.

This is the first demonstration that a preference for the task of deciphering number symbols can develop in the ventral temporal cortex regardless of any visual experience. This is quite intriguing especially in light of previous discussions of the origins of category specificity for letters and faces that have hypothesized a role for visual parameters such as visual frequency, foveal projections or a preference for line junctions^19^,^20^,^21^. This implicit hypothesis is also apparent in the conventional name for those areas, for example, VNFA. Quite remarkably, however, we show a preference for number symbols in the blind following as little as ~30 h of training on decoding shapes and colours using the EyeMusic SSD and only brief training on number symbols, that is, on the stimuli used in the experiment.

We suggest that the role of this area is to decipher the shapes of numerals, which are symbols arbitrarily defined by culture, for the purpose of accessing the corresponding quantity representation through the connections to the intraparietal cortex (discussed below). Our results suggest that the task performed by the ventral temporal cortex remains even when the shape of the symbols is encoded using a non-visual sensory modality and, even more interestingly, without any exposure to vision during development. This is in accordance with previous results from our team suggesting that there is nothing visual about the VWFA because it performs the task of deciphering letter symbols for the purpose of accessing the corresponding phoneme regardless of the sensory modality used for symbol input^27^,^28^. These two results join similar evidence from various teams showing preference for non-visual input in what is classically referred to as the visual cortex. Among the preponderant examples are: the task performed in part of the lateral occipital complex, which is to decipher the geometrical shape of objects^26^,^46^,^47^, the task performed in the middle occipital gyrus, which is to detect locations^29^ and the task performed in hMT+, which is to process motion^34^,^36^.

The question that follows would be: why then do so many studies show visual processing in these areas? We suggest that this specialization for vision emerges only because, in sighted individuals, vision is the dominant modality that provides most of the needed information for performing the mentioned tasks. However, when information is provided using different sensory modalities, the same preference can develop even without any visual experience. This conclusion corresponds to our recent suggestion on the organization of the cortex as a set of computation-specific systems, characterized by the task they perform, rather than sensory-specific systems^23^,^48^. This suggestion is in-line with the theories of the metamodal/supramodal organization of the brain^24^,^49^.

No significant group effect was evident in the random-effects analysis when contrasting the letter identification task versus the other tasks. This is in contrast to previous studies in the congenitally blind that showed activation of the VWFA for tactile Braille stimulation when contrasting Braille words versus nonsense Braille^27^ and for SSD auditory stimulation when contrasting letters versus other visual categories^28^ (for example, faces and houses). Inspecting our data using a fixed-effects analysis with strict correction for multiple comparisons showed one activated area in the visual cortex overlapping the VWFA (Supplementary Fig. 4) suggesting that there is a trend towards preference for the letter identification task. This difference between our study and previous studies is possibly due to the little experience subjects had with reading using the EyeMusic SSD, especially in comparison with studies using Braille where subjects have years of experience^27^ and SSD studies that conducted dedicated training for reading^28^. Furthermore, both mentioned studies did not contrast reading with a control condition that contained exactly the same symbols but either used nonsense symbols or other visual categories. Therefore, the difference could also be attributed to the nature of the identification task and the use of the same stimuli under all tasks. It would be plausible to suggest that in the current study, all tasks implicitly activated the VWFA because subjects could not refrain from extracting the Latin letter from the encoded shape and the stronger activation for the letter identification task was less apparent. Indeed, an inspection of the data shows that the VWFA was activated versus rest under all three tasks (*Numeral*, *Colour* and *Letter*).

Having seen the distinct cortical areas for number symbols (VNFA) and letters (VWFA), we now discuss how they might have emerged. Number symbols and letters are, arguably, highly similar in their physical features (consider for instance the symbol for zero and the letter O in English). It is only a cultural convention that makes them different. In fact, some cultures used the exact same symbol as a number with an assigned quantity and as a letter with an assigned phoneme, for example, in Roman script V is used to represent both the letter V and the number 5. The emergence of an area with preference for the task of deciphering number symbols, which is distinct from the area with preference for the task of deciphering letters, and yet located at a systematically reproducible localization across subjects (whether blind or sighted), cannot be explained by the visual features of such symbols. It is more plausible, however, to propose that cortical connections between these areas and networks processing relevant information play a role in this emergence. Therefore, we hypothesized that an area showing preference for number symbols could arise at a reproducible anatomical location across subjects if such a region showed systematic connections to evolutionarily ancient cortical areas of the intraparietal cortex responsible for the representation and mental manipulation of quantities^40^. Similarly, an area showing preference for letters could arise at a reproducible anatomical location across subjects if such a region showed systematic connections to cortical areas responsible language processing^50^,^51^. Our data confirm these hypotheses by (1) showing a preference for numerals that is reproducible across subjects, different from that of letters, even when using the exact same stimuli, thus ruling out the effect of physical differences; (2) showing functional connectivity between this area and a site in the intraparietal sulcus, which is activated whenever numerical quantities are processed; (3) showing, conversely, functional connectivity between the VWFA and language-processing areas; (4) showing that these connectivity patterns do not arise from mere hemisphere-specific differences.

In addition, these results are in agreement with the theory of cultural recycling^52^, which suggests that the acquisition of novel cultural inventions is only feasible inasmuch as it capitalizes on prior anatomical and connectional constraints and invades pre-existing brain networks capable of performing a function sufficiently similar to what is needed by the novel invention. In addition, other factors such as the specifics of how literacy and numeracy are learned, as well as the distinctive functions of numerals and letters in our education and culture, could also account for the segregation of their preferences. In the future, it would be very important to provide a stricter test of the present ideas by measuring anatomical and functional connectivity in young children (blind and sighted) before symbol acquisition, and see if this connectivity predicts the future localization of the VWFA and VNFA.

We close by noting that the EyeMusic SSD itself, much like the invention of writing, provided a novel mode of input into the language and number systems, and thus may be seen as an original cultural invention that, after a few weeks of training, ‘recycles’ the shape recognition networks of the visual cortex. We suspect that brain plasticity still offers a huge potential for innovation in this relatively new domain of technological devices.

## Methods

### Subjects

The study included nine blind subjects (two male, mean age: 34±3) and twelve sighted subjects (six male, mean age 30±10). The blind group consisted of seven congenitally blind subjects, a subject blinded by the age of 6 months, and a subject who had congenital blindness in the left eye and had lost sight in the right eye by the age of 1 year. One congenitally blind subject has faint light perception without the ability to localize light or recognize any form or shape. For a detailed description of blindness characteristics, please refer to Supplementary Table 1. In addition to the reported results, we ran all the analyses on the subgroup of seven congenitally blind subjects and no difference was found in the main findings. Subjects were native Hebrew speakers, all read Braille in both Hebrew and English. We initially scanned 15 sighted subjects out of which three were excluded due to head movement. All subjects reported normal hearing with no known neurological conditions. The Tel-Aviv Sourasky Medical Center Ethics Committee approved the experimental procedure and written informed consent was obtained from each subject. Braille forms were used for the blind subjects. The training procedure was approved by the Ethics Committee of the Hebrew University of Jerusalem, Israel.

### Visual stimulation for the blind using sensory substitution

We used the EyeMusic SSD, which recruits music to represent visual information. The visual-to-auditory algorithm is based on the image sweep-line technique and each image is processed column-by-column from left to right constructing a ‘soundscape’: a combination of sounds that represents this image^53^. Given an image, the algorithm resizes it, colour-clusters its pixels to get a six-colour image and then creates its spatially preserving auditory representation (Fig. 1a). The two-dimensional (2D) image’s *x* axis is mapped to the time domain, that is, pixels situated on the left side of the image will sound before the ones situated on its right side. The *y* axis is mapped to the pitch domain, that is, pixels situated on the upper side of the image will sound higher in frequency, while those on its lower side will sound lower in frequency. The colour information is represented by the timbre of the different instruments (see ref. 41 for a complete description and samples).

### Training procedure

In order to familiarize the subjects with the SSD algorithm, a 15-lesson training programme was constructed. The goal of the programme was to teach the ability to interpret basic geometrical shapes in the colours available to the system (white, red, green, blue and yellow; Supplementary Fig. 10). Each lesson used a batch of images in five colours. The number of images per lesson ranged from 7 to 20, reaching a total of 1,090 images in the whole training procedure. The lessons started with basic horizontal and vertical lines in a variety of thicknesses and locations on the image. As lessons advanced, lines in different orientations were added and shapes were gradually constructed. The shapes included various line combinations and geometrical shapes such as triangles, squares and circles. The last lessons focused on the combinations of various shapes in the same image with varying locations. The English letters H, I, N, F, E, M and X were utilized to demonstrate the several combinations of horizontal, vertical and diagonal lines. The emphasis when training on these letters was primarily on their geometric features, while their phonetic representations were only briefly mentioned at the very end when subjects could fully recognize the shape. Each subject was trained 25–30 h. An examination of colour knowledge was performed after each five lessons with near-ceiling success for all subjects.

### Experiment design

We designed a slow event-related fMRI paradigm with three tasks. The same physical stimuli were used under all tasks. The difference between tasks was in the information to be extracted from the stimuli. We used the EyeMusic SSD to generate the auditory representation of the symbols I,V and X in white, blue and red, leading to nine stimuli in total. For each stimulus, subjects had to identify either (1) which Roman numeral it represented (*Numeral* task), (2) the colour of the symbol (*Colour* task) or (3) which Latin letter it represented (*Letter* task). The subjects had to respond by pressing one out of three buttons. Before the experiment and outside the scanner, subjects were instructed on the button mapping of the possible responses in each task.

Each trial started with a 0.4-s auditory instruction followed by 1.6 s of silence. The instructions were a natural voice recording in Hebrew of the words ‘Numeral’, ‘Colour’ and ‘Letter’, and were used to announce the upcoming task. Then the recorded stimulus soundscape was played three times over a period of 10 s, 3.3 s for each playback. The subjects were instructed to listen carefully to the soundscapes and to extract the needed information according to the task. A ‘ding’ sound that signalled the start of a 3-s response period was played immediately afterwards. A rest period of 9 s was given between trials (see Fig. 1b for design and sample trials).

### Task training

Each subject was trained on performing the three tasks of the experiment, namely extracting the Roman numeral, Latin letter and colour of each stimulus used in the experiment. This training was conducted in a dedicated session lasting ~1 h, after the end of the training procedure described above and before the fMRI acquisition. Near-ceiling performance was achieved by all subjects, ensuring that the three blocks of the experimental design were balanced for training and difficulty.

### fMRI protocol

Each blind subject underwent three fMRI runs, the first being a 10-min acquisition of resting-state spontaneous BOLD signal fluctuations^54^ followed by two pseudorandomly ordered sequences of 27 experiment trials each. Every stimulus appeared three times, once under each task, leading to nine repetitions per task. The scanning order of the two sequences was counterbalanced between subjects. Task performance was recorded. The sighted subjects only participated in the resting-state acquisition and were blindfolded throughout the acquisition.

### Technical details

The experiment was presented using the software package Presentation (Neurobehavioral Systems, Albany, CA, USA). The auditory stimulation was presented to both ears using fMRI-compatible headphones (OptoACTIVE system, Optoacoustics Ltd, Israel). Subjects were provided with earplugs to further reduce the scanner noise. The subjects were presented with the auditory instructions, the stimuli and the ding sound inside the scanner before the experiment, with all insulations in place.

### Behavioural data analysis

Single-subject behavioural results are reported in Supplementary Table 2. Wilcoxon rank-sum test was used for assessing whether the performance levels were above the chance level. There were always three possible answers and the chance level was thus 1/3. One-way ANOVA was used to assess whether performance differed across tasks.

### MRI acquisition

The BOLD fMRI measurements were performed in a whole-body 3T GE Signa scanner (GE Medical Systems, USA). The gradient-echo EPI pulse sequence was used.

For the slow event-related experiment, we acquired 27 slices of 4.5 mm thickness and 0 mm gap. The data in-plane matrix size were 64 × 64, field of view (FOV) 220 mm × 220 mm, time to repetition (TR)=1.5 s, flip angle=70° and TE=35 ms. In all, 458 and 459 whole-brain images were collected during the two experiment sequences, respectively. The first 10 images of each scan were excluded from the analysis because of non-steady-state magnetization. Three runs were discarded due to technical difficulties with the sound system.

For the resting-state run, we acquired 46 slices of 3 mm thickness and 0 mm gap. The data in-plane matrix size were 64 × 64, FOV 240 mm × 240 mm, TR=3 s, flip angle=90° and time to echo (TE)=30 ms. In all, 200 whole-brain images were collected during resting-state run. The first two images of each scan were excluded from the analysis because of non-steady-state magnetization, and the last 18 images were excluded to conform to previously acquired data.

*Anatomy*. Three-dimensional T1-weighted anatomical volumes were collected. 3D-Spoiled Gradient Recalled (SPGR) echo sequence was used with typical parameters of FOV 23 cm (RL) × 23 cm (VD) × 17 cm (AP), TR=9 ms, TE=4 ms, flip angle=13°, data matrix: 160 × 160 × 144 zero-filled to 256 in all directions, that is, ~1 mm isovoxel native data.

### Data analysis

Data analysis was performed using the Brain Voyager QX 2.6 software package (Brain Innovation, Maastricht, the Netherlands) and NeuroElf toolbox version 0.9c ( http://neuroelf.net/) for MATLAB (MathWorks, Natick, MA, USA). All fMRI data went through the standard preprocessing procedures that consisted of slice scan time correction, 3D motion correction and high-pass filtering using the GLM approach with Fourier basis set (cutoff: two cycles per scan). No data included in this study showed motion exceeding 2 mm in any given axis of the 3D head motion correction, except for one run in the slow-event-related task that did not exceed 3 mm. Functional and anatomical data sets for each subject were aligned and fit to standardized Talairach space^55^. Each set of anatomical images in Talairach space underwent the automatic white-matter grey-matter segmentation functions embedded in BrainVoyager and cortex masks were created for each brain. External voxel coordinates were translated from Montreal Neurological Institute (MNI) space to Talairach space according to the Nonlinear Yale MNI to Talairach Conversion Algorithm^56^.

For the slow event-related experiment, single subject data were spatially smoothed with an 8-mm 3D Gaussian in order to reduce intersubject anatomical variability. BOLD intensity thresholding was performed to ensure that the voxels analysed are not influenced by signal dropout and do not contain attenuated signal, which could lead to false-positive results (detailed below). The data were then grouped using a GLM using a predictor for each task (Numeral, Colour and Letter; Fig. 1b) in a hierarchical random-effects analysis^57^. Serial correlations were corrected using a voxel-wise AR(2) model^58^.

In order to determine the whole-brain preferential activation for the *Numeral* identification and the *Letter* identification tasks, we used the *Task*-versus-others contrast in conjunction with the *Task*-versus-rest contrast. We refer to these conjunctions as the *Task name* contrast, for example, *Numeral* task contrast, throughout the text. We used the ‘Minimum Statistic compared to the Conjunction Null’ method^59^ (MS/CN) therefore, refuting the null hypothesis for the conjunction infers a logical AND, which means that both comparisons are satisfied.

We also contrasted the *Numeral* task with the *Letter* task alone using the *Numeral*-versus-*Letter* and *Numeral*-versus-rest conjunction (MS/CN^59^) to show that the area with preferential activation for number symbols does not only arise when compared with the *Colour* task. In all cases, the minimum significance level was set to *P*<0.05 corrected for multiple comparisons using the spatial extent method based on the theory of Gaussian random fields^60^,^61^. This method takes the data contiguity of neighbouring voxels directly into account and corrects for the false-positive rate of continuous clusters (a set-level statistical inference correction). This was performed based on the Monte Carlo simulation approach, extended to 3D data sets using the Cluster-level Statistical Estimator plug-in for BrainVoyager QX and using a cortex mask that contained all the voxels present in at least five cortices, that is, the majority of cortices.

In case of the *Letter* contrast, the random-effects group analysis did not yield any significant voxels; therefore, we grouped the subjects using a GLM fixed-effects analysis with serial correlations were corrected using a voxel-wise AR(2) model^58^. The minimum significance level was set to *P*<0.05 corrected for multiple comparisons using the strict Bonferroni family-wise correction approach.

In order to verify that it is not the mere instruction to think about Numerals, Colours and Letters is what induces the activations observed, we added a control analysis that splits each event into three parts: (1) Instruction (lasting for 1.5 s); (2) Stimulus (lasting for 10.5 s); (3) Response (lasting for 3 s; Supplementary Fig. 3A). We therefore defined three predictors per task according to this division for the GLM analysis with all other aspects were maintained as described above. We then used the *Numeral*-versus-others contrast in conjunction (MS/CN^59^) with the *Numeral*-versus-rest contrast for each of the three parts of the protocol, for example, ‘Numeral instruction’-versus-‘other instructions’. This enabled disentangling between the activation induced by each of the three parts of the events.

For the resting-state analysis, ventricle and white-matter average time courses were calculated per subject and regressed out of the spontaneous BOLD data using MATLAB, followed by a bandpass filter of 0.01–0.1 Hz (frequencies in which spontaneous BOLD fluctuations occur^54^,^62^). Single subject data were spatially smoothed with a 6-mm 3D Gaussian in order to reduce intersubject anatomical variability. BOLD intensity thresholding was performed to ensure that the voxels analysed were not affected by signal dropout and did not contain attenuated signal, which could lead to false-positive results (detailed below). A region of interest (ROI) was defined as the peak functional voxel of the activation in the random-effects analysis under the *Numeral* task, and is referred to as the *Numeral seed.* A second ROI was defined as the peak functional voxel of the activation in the fixed-effects analysis under the *Letter* task, and is referred to as the *Letter seed.* Individual average time courses were sampled from these seed ROIs, *z*-normalized and then each time course was used as a single-subject predictor in a group analysis using GLM in a hierarchical random-effects analysis^57^. We used partial correlation, that is, regressing out seed A from the data and the GLM predictor when running the GLM for seed B, as implemented by the dummy predictor feature of BrainVoyager. This was performed to make sure that the correlations observed are specific to the time course of the ROI we are interested in, and to ensure that the connectivity patterns are not due to shared signal between the ROIs. Partial correlation has been previously used in the resting-state fMRI literature with similar aims of improving the specificity of functional connectivity^63^,^64^,^65^,^66^ and is considered among the standard methods^67^. The minimum significance level was set to *P*<0.05 corrected for multiple comparisons using the spatial extent method (described above). The exact same analysis was repeated using the Pearson product–moment correlation coefficient instead of the partial correlation coefficient in order to verify that the different results between the two seeds are not a mere artifact of the use of partial correlation (see Supplementary Fig. 7).

Furthermore, in order to explore brain preference between the two seeds, we computed the following contrast maps: (1) *Numeral seed* versus *Letter seed* in conjunction (MS/CN^59^) with the *Numeral seed* map; (2) *Letter seed* versus *Numeral seed* in conjunction (MS/CN^59^) with the *Letter seed* map. This was performed by extracting the single subject beta-values resulting from the *Numeral seed* and *Letter seed* group analysis described above and computing a voxel-wise paired *t*-test between them. Afterwards, the Seed A>Seed B map was masked by the Seed A>0 map. The minimum significance level was set to *P*<0.05 corrected for multiple comparisons using the spatial extent method (described above). Here as well, we repeated the same analysis using the Pearson product–moment correlation coefficient instead of the partial correlation coefficient in order to verify that the different results between the two seeds are not a mere artifact of the use of partial correlation (see Supplementary Fig. 8).

To ensure that the maps with co-activation for the *Numeral* and *Letter* seeds do not arise because of the fact that the *Numeral* seed is in the right hemisphere and the *Letter* seed is in the left hemisphere, we repeated the analysis described above using symmetrical seeds. For each of the original seeds, we defined its symmetrical seed by changing the sign of its *x* coordinate. We therefore performed the analysis on the *Numeral seed* and the *Sym. Letter seed* pair situated in the right hemisphere and on the *Sym. Numeral seed* and the *Letter seed* pair situated in the left hemisphere.

### Result representation

Average BOLD signal percent change plots are computed using the event-related averaging feature in BrainVoyager by point-by-point averaging of time course segments belonging to the same condition. The graphs generated reflect the average time courses for each condition across voxels belonging to a single ROI across subjects.

A 3D surface reconstruction was performed on one set of anatomical images in Talairach space using the Brain Voyager QX 2.6 software. The images went through inhomogeneity correction followed by the automatic white-matter grey-matter segmentation functions embedded in BrainVoyager. The surface was then inflated to create a Talairach-normalized representative cortical surface on which the activation maps were superimposed.

### BOLD intensity thresholding

Among the artifacts present in fMRI data, a significant signal dropout can be observed in areas with tissue/air interfaces and is because of susceptibility artifacts^68^,^69^,^70^. This signal dropout is of special relevance to our data set because the area in the rITG found in ref. 10 has been confirmed to fall close to or within an fMRI dropout zone, which lies above the ear canal (Shum *et al*. (Fig. 5))^10^. Therefore, we developed a method for thresholding the BOLD signal that ensures the exclusion of voxels with attenuated signal intensity. We first generate a histogram of the maximum BOLD signal intensity values per voxel for each run (Supplementary Fig. 11—red circles). The resulting histogram can be modelled as a combination of two Gaussian distributions and a linear trend, where the first Gaussian represents low-intensity voxels (signal dropout or no brain signal), and the second Gaussian represents high-intensity voxels (white- or grey matter; Supplementary Fig. 11—blue line). An intensity threshold separating high- and low-intensity voxels was set to exclude 99% of the voxels under the first distribution, that is, voxels with low-intensity signal. All voxels with signal intensity value below the threshold were masked and therefore excluded from the analysis.

## Additional information

**How to cite this article**: Abboud, S. *et al*. A number-form area in the blind. *Nat. Commun.* 6:6026 doi: 10.1038/ncomms7026 (2015).

## Supplementary Material

Supplementary Information Supplementary Figures 1-11, Supplementary Tables 1-5, Supplementary Reference

## Figures and Tables

**Table 1:** Areas with preferential activation for the *Numeral* versus the *Colour* and *Letter* tasks.

**Brain areas**	**Talairach coordinates**						
	**Hemi.**	**Size**	**Peak** ***x***	**Peak** ***y***	**Peak** ***z***	***t*** **(8)**	***P***
Inferior temporal gyrus	R	2,665	53	−44	−12	4.2915	0.0026

The *t*-test results, reflecting the random-effect group analysis (*n*=9), on the contrast of the *Numeral* versus the *Colour* and *Letter* tasks in the peak of the area in the inferior temporal gyrus.
