# Supplementary material for: A number-form area in the blind
Source: Nat Commun. 2015 Jan 23;6:6026. doi: 10.1038/ncomms7026 (PMC4338545; doi:10.1038/ncomms7026)
Supplement: Supplementary Information — Supplementary Figures 1-11, Supplementary Tables 1-5, Supplementary Reference [file ncomms7026-s1.pdf]

**Supplementary Information**

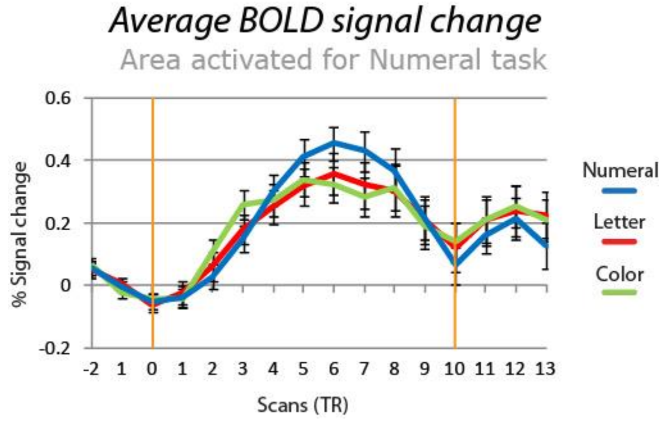


**Supplementary Figure 1 – Average BOLD percent signal change –** average time-course of percent signal change across trails (9 per task) and subjects (n=9) in the area with preferential activation to the *Numeral* task in the rITG. The *Numeral* task trials are in blue, *Letter* task trials in red, and *Color* task trials in green, error bars represent the standard error of the mean (SEM). The first orange vertical line marks the beginning on the trial and the second orange line marks the end of the trial.

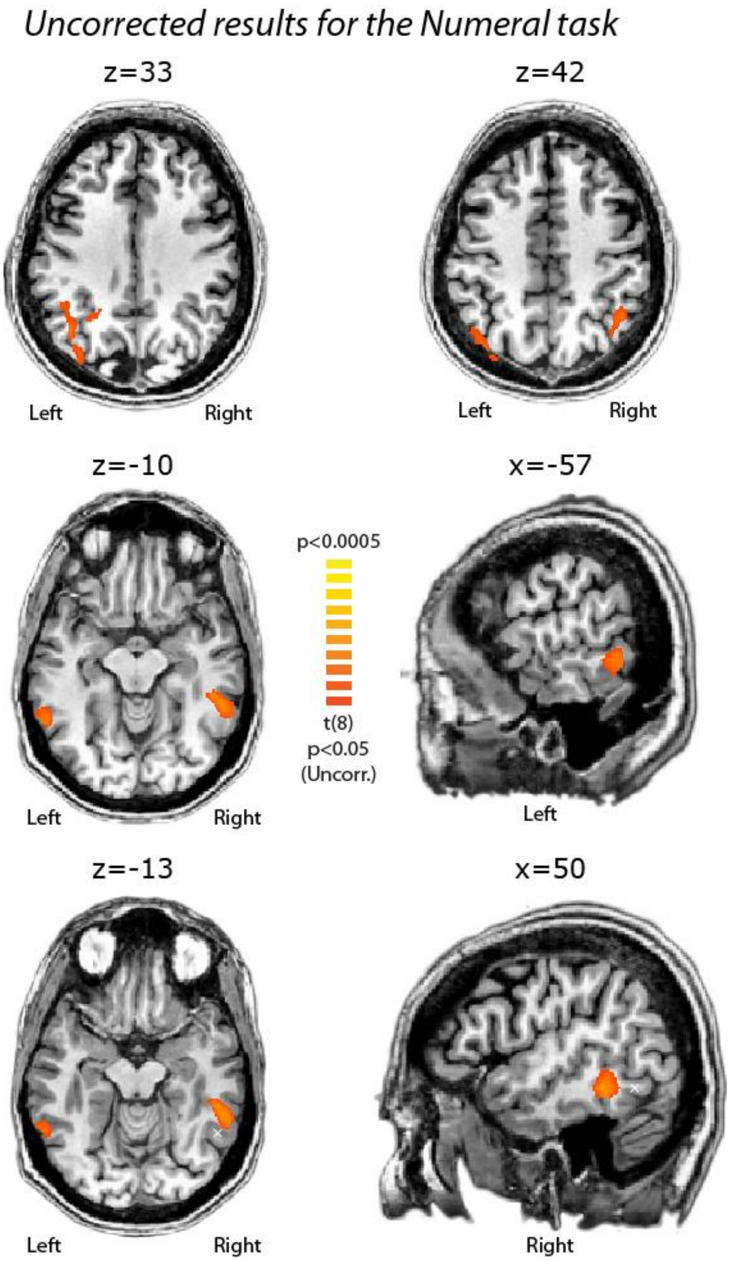


**Supplementary Figure 2 – Preferential activation for the *Numeral* task uncorrected –** the result of the random-effects group analysis (Not corrected for multiple comparisons) for the contrast between the *Numeral* task versus the *Color* and *Letter* tasks. In addition to the cluster in the right ITG (slices z=-10, -13 and x=50), we observe a cluster in the symmetrical area of the left ITG (slices z=-10, -13 and x=-57) and a bilateral activation in the intraparietal sulcus (slices z=33, 42). The white crosses indicate the location of the peak of the activation found by Shum et al. (2013 (Ref. 1); x=-53, y=-44, z=-12).

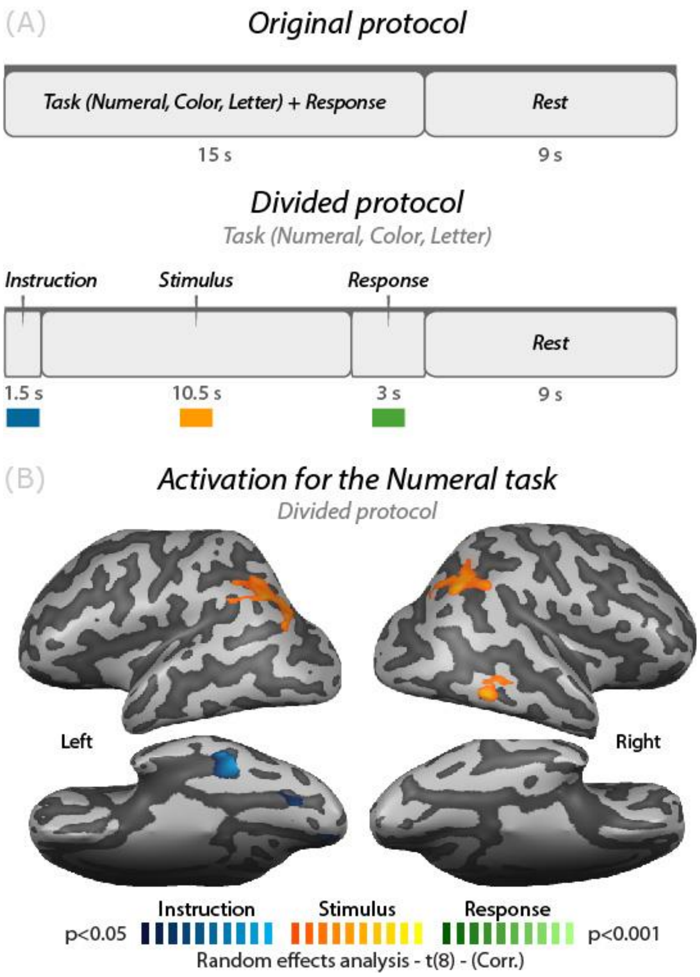


**Supplementary Figure 3 – Preferential activation for the *Numeral* task in the blind under the divided protocol – (A)** First row: the original analysis protocol. Second row: a protocol dividing each event into three stages, i.e., instruction, stimulus and response. **(B)** The result of the random-effects group analysis (corrected for multiple comparisons) in a group of blind subjects (n=9) for the *Numeral* task versus the *Color* and *Letter* tasks using the 3 separate predictors corresponding to the protocol in (A). In shades of blue, the result map of the contrast between the *Numeral* task and the other tasks during the instruction period. In red-to-yellow, the result map of the contrast between the *Numeral* task and the other tasks during the stimulus period. In red-to-The contrast between the *Numeral* task and the other tasks during the response period did not show any significant clusters. It can be seen that the mere instruction to think about numerals does not lead to the activation reported in the VNFA.

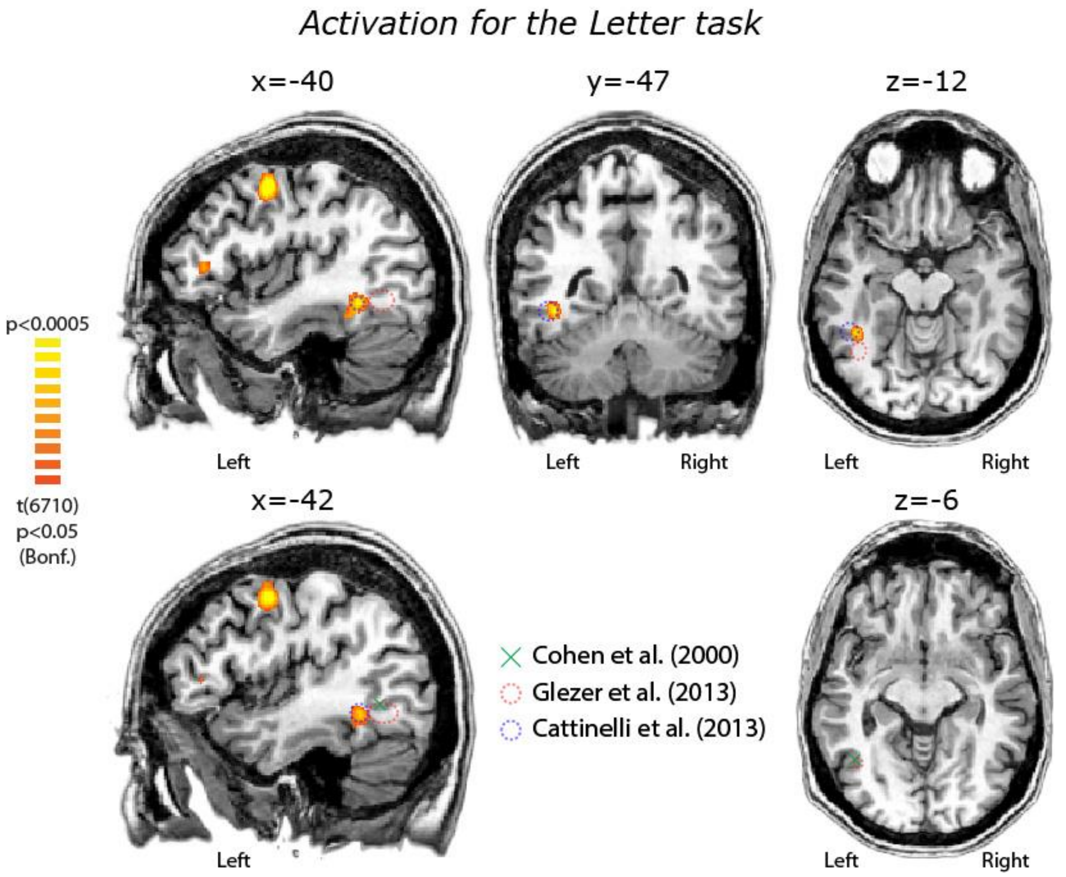


**Supplementary Figure 4 – Preferential activation for the *Letter* task –** the result of the fixed-effects group analysis (Bonferroni corrected for multiple comparisons, p<0.000003 uncorrected) for the contrast between the *Letter* task v   ersus the *Color* and *Numeral* tasks. Sagittal, coronal and transverse slices show the activation in the VWFA which resides in the occipitotemporal sulcus in addition to activations in the superior frontal gyrus and middle frontal gyrus (See Supplementary Table 5 for the full list of areas). Several coordinates of the VWFA as found in previous studies are indicated on the figure. A green cross indicates the peak of activation as reported by Cohen et al. (2000 (Ref. 2); peak coordinates x=-42, y=-57, z=-6), a dashed red circle marks the mean coordinates as reported by Gelzer et al. (2013 (Ref. 3); mean coordinates x=--40, y=-59, z=-10 – SD x=5, y=8, z=6) and a dashed blue circle marks the mean activation in a meta-analysis by Cattinelli et al. (2013 (Ref. 4); Table 4; mean coordinates x=-45, y=-47, z=-12 – mean SD=6).

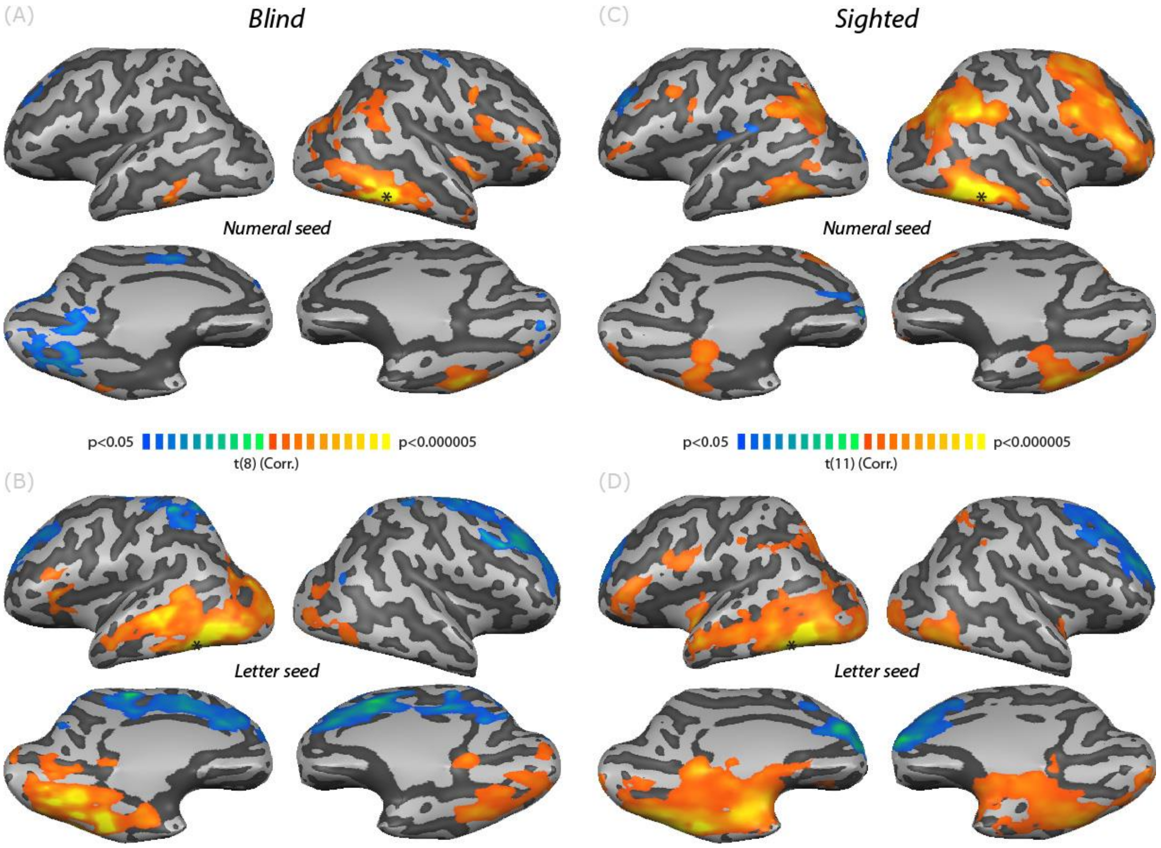


**Supplementary Figure 5 – Functional connectivity –** the result of the random-effects group analysis (corrected for multiple comparisons) of functional connectivity to the seeds defined by (1) the peak of the area active for the *Numeral* task, denoted as the *Numeral seed* and (2) the peak of the area active for the *Letter* task, denoted as the *Letter seed*. Lateral and medial views of an inflated brain overlaid with the connectivity maps and an asterisk marking the relevant seed. *Blind group (n=9)* functional connectivity maps from the *Numeral seed* **(A)** and from the *Letter seed* **(B)**. *Sighted group (n=12)* functional connectivity maps from the *Numeral seed* **(C)** and from the *Letter seed* **(D)**. Positive co-activation with the seed is illustrated by the red to yellow range and negative co-activation with the seed is illustrated by the blue to green range. Both groups show a co-activation of the *Numeral seed* with areas involved in quantity processing and the *Letter seed* with areas involved in language processing. This figure expands Fig. 3 in the text by adding negative co-activations and a medial view.

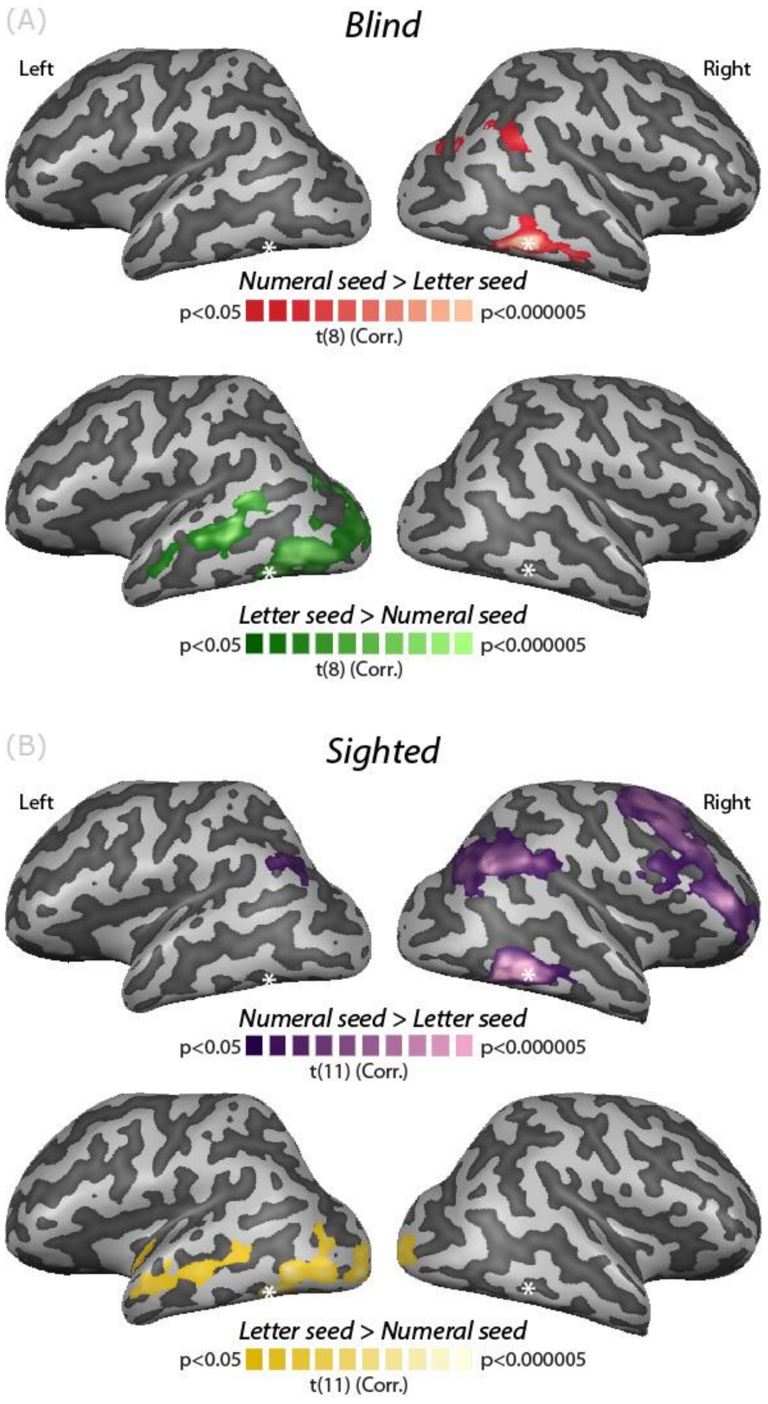


**Supplementary Figure 6 – Functional connectivity contrast –** the result of the functional connectivity group contrast analysis (corrected for multiple comparisons) between the *Numeral seed* and the *Letter seed* overlaid on the lateral view of an inflated brain. A white asterisk in the right hemisphere marks the *Numeral seed* and a white asterisk in the left hemisphere marks the *Letter seed*. The contrast is computed by the conjunction of Seed A > Seed B and Seed A > 0 (Further detailed in Methods). **(A)** *Blind group (n=9)* – the areas with preference for the *Numeral seed* are shown in red and the areas with preference for the *Letter seed* are shown

in green. **(B)** *Sighted group (n=12)* – the areas with preference for the *Numeral seed* are shown in purple and the areas with preference for the *Letter seed* are shown in yellow.

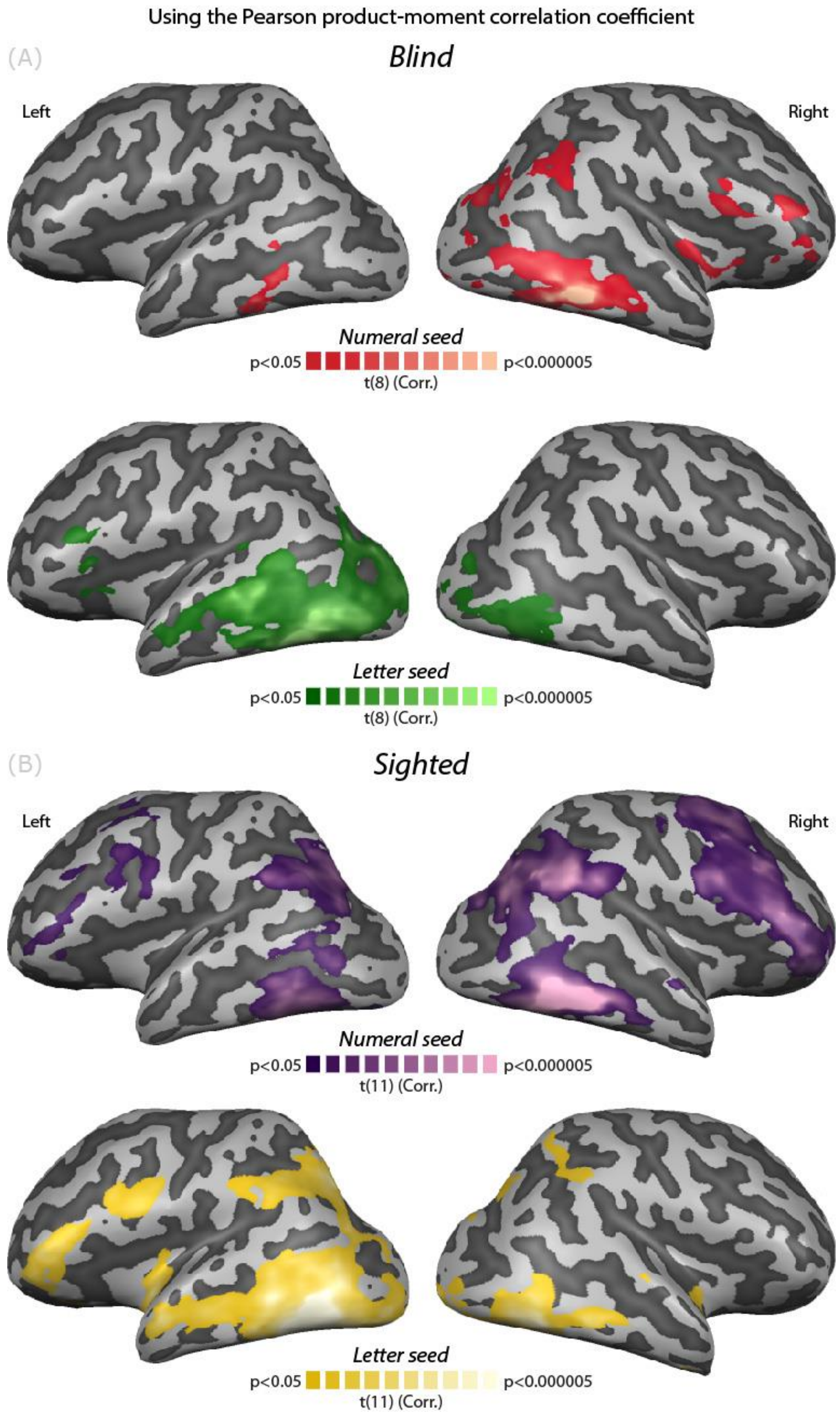


**Supplementary Figure 7 – Functional connectivity results using the Pearson product-moment correlation coefficient –** the result of the random-effects group analysis (corrected for multiple comparisons) of functional

connectivity to the seeds defined by (1) the peak of the area active for the *Numeral* task, denoted as the *Numeral seed* and (2) the peak of the area active for the *Letter* task, denoted as the *Letter seed*. This figure shows a similar result to Fig. 3, while using the Pearson product-moment correlation coefficient instead of the partial correlation coefficient. All other analysis parameters were kept constant.

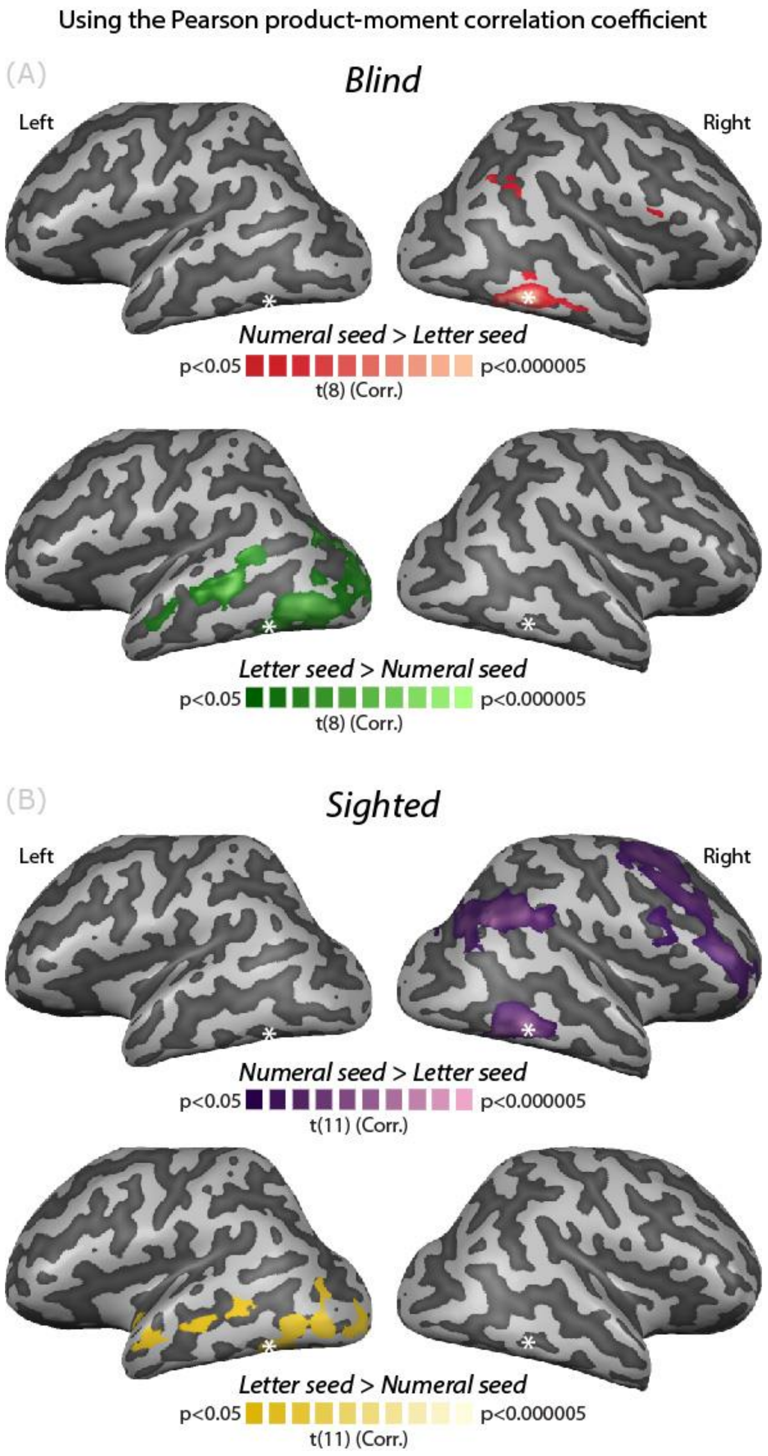


**Supplementary Figure 8 – Functional connectivity contrast using the Pearson product-moment correlation coefficient –** the result of the functional connectivity group contrast analysis (corrected for multiple comparisons) between the *Numeral seed* and the *Letter seed* overlaid on the lateral view of an inflated brain. A white asterisk in the right hemisphere marks the *Numeral seed* and a white asterisk in the left hemisphere marks the *Letter seed*. The contrast is computed by the conjunction of Seed A > Seed B and Seed A > 0 (Further detailed in Methods). This figure shows a similar result to Supplementary Fig. 6, while using the Pearson

product-moment correlation coefficient instead of the partial correlation coefficient. All other analysis parameters were kept constant.

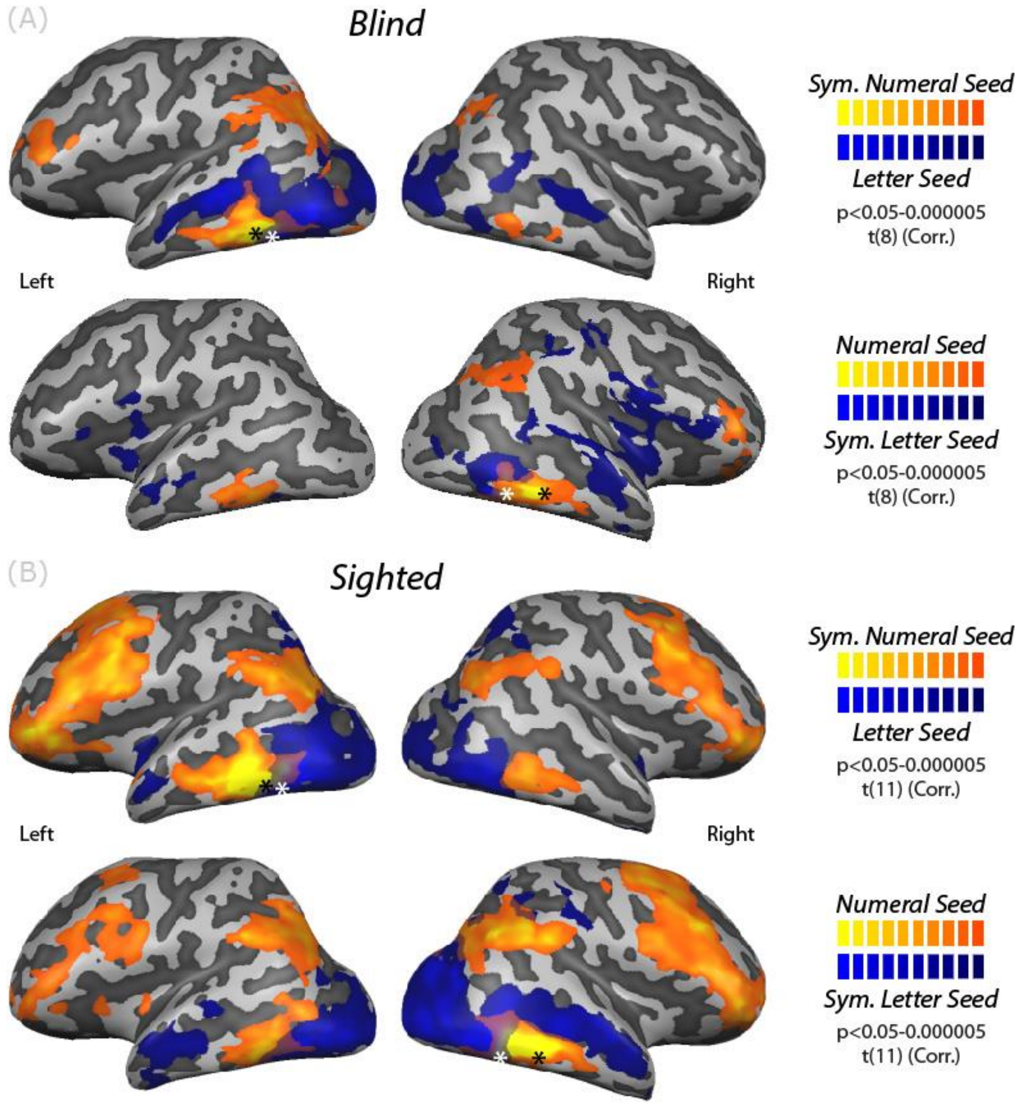


**Supplementary Figure 9 – Functional connectivity control –** the result of the random-effects group analysis (corrected for multiple comparisons) of functional connectivity to the seeds defined by (1) the peak of the area active for the *Numeral* task, denoted as the *Numeral seed*, (2) its symmetrical seed defined by changing the sign of the x-coordinate, denote as the Sym. Numeral seed, (3) the peak of the area active for the *Letter* task, denoted as the *Letter seed* and (4) its symmetrical seed defined by changing the sign of the x-coordinate, denote as the *Sym. Letter seed*. Lateral view of an inflated brain overlaid with the connectivity maps. The numeral seeds are marked by a black asterisk and with functional connectivity maps in red to yellow, the letter seeds are marked by a white asterisk with functional connectivity maps in dark-blue to light-blue. **(A)** *Blind group (n=9)*, **(B)** *Sighted group (n=12)*.

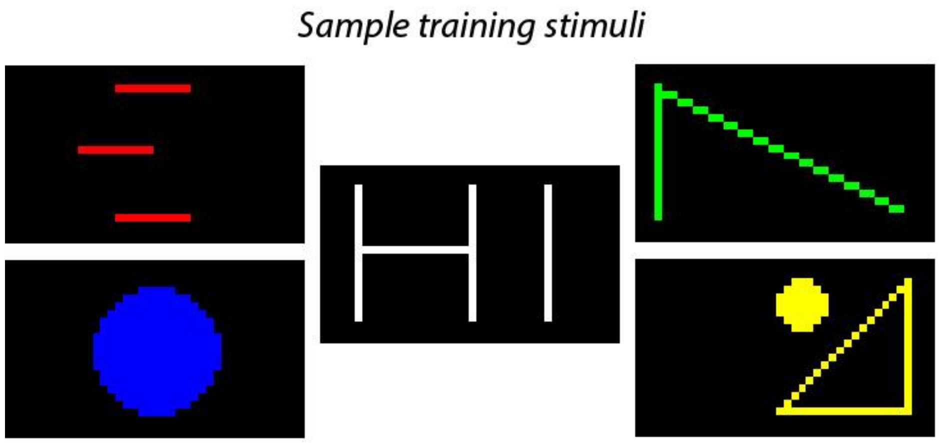


**Supplementary Figure 10 – Training procedure stimuli –** Sample stimuli used during the training procedure in the five colors of the algorithm for training on shape and color recognition abilities.

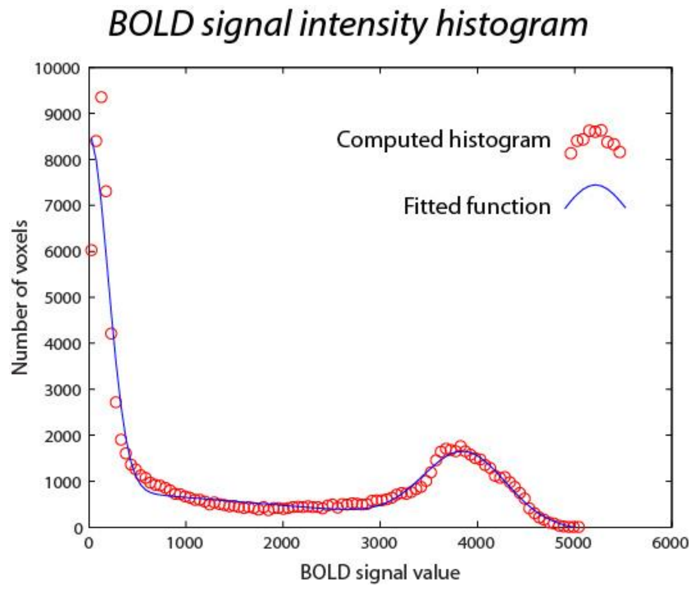


**Supplementary Figure 11 – BOLD signal intensity histogram –** A histogram of BOLD signal intensity values in a representative subject when using 100 bins (Red circles). The maximal signal value throughout the acquisition was used for each voxel. A function with two Gaussian distributions and a linear trend is fitted to the histogram (Blue line, r=0.97 is the correlation coefficient between the histogram and the fitted function in the plot above). This fitted function, evaluated per each run of each subject, is then used to determine the BOLD signal intensity cutoff value. Voxels with intensity values under the calculated cutoff are eliminated from the analysis to avoid including voxels with signal dropout.

**Supplementary Table 1 – Causes of blindness**

| *#* | *Age* | *Gender* | *Cause of blidness* | *Onset* | *Light perception* |
|---|---|---|---|---|---|
| *1* | 24 | F | Microphthalmia, Retinal detachment | **1 yr** | None |
| *2* | 30 | F | Microphthalmia | 0 | None |
| *3* | 31 | F | Retinopathy of prematurity | 0 | None |
| *4* | 38 | F | Retinoblastoma | **6 mo** | None |
| *5* | 42 | M | Retinopathy of prematurity | 0 | None |
| *6* | 38 | M | Retinopathy of prematurity | 0 | None |
| *7* | 35 | F | Enophthalmos | 0 | None |
| *8* | 30 | F | Leber congenital amaurosis | 0 | **Faint** |
| *9* | 37 | F | Microphthalmia | 0 | None |

**Supplementary Table 2 – Single-subject behavioral results**

| *#* | *Color* | *Numeral* | *Letter* |
|---|---|---|---|
| *1* | 83% | 83% | 94% |
| *2* | 100% | 94% | 100% |
| *3* | 89% | 78% | 100% |
| *4* | 89% | 33% | 56% |
| *5* | 94% | 100% | 89% |
| *6* | 89% | 100% | 89% |
| *7* | 89% | 94% | 94% |
| *8* | 89% | 100% | 100% |
| *9* | 94% | 94% | 78% |

**Supplementary Table 3 – Areas with preferential activation for the *Numeral* versus the *Letter* task random-effects analysis**

| | | | Talairach coordinates | | | | |
|---|---|---|---|---|---|---|---|
| *Brain areas* | *hemi.* | *size* | *peak x* | *peak y* | *peak z* | *t(8)* | *p* |
| *Inferior temporal gyrus* | R | 2066 | 50 | -35 | -12 | 3.3988 | 0.0093 |

**Supplementary Table 4 – Areas with preferential activation for the *Numeral* versus the *Color* and *Letter* tasks – random-effects analysis, not corrected for multiple comparisons**

| | | | Talairach coordinates | | | | |
|---|---|---|---|---|---|---|---|
| *Brain areas* | *hemi.* | *size* | *peak x* | *peak y* | *peak z* | *t(8)* | *p* |
| Cluster 1<br>*Inferior temporal gyrus* | R | 2665 | 53 | -44 | -12 | 4.291544 | 0.002646 |
| Cluster 2<br>*Inferior temporal gyrus* | L | 1176 | -55 | -53 | -9 | 3.569318 | 0.007301 |
| Cluster 3<br>*Intraparietal sulcus* | R | 567 | 44 | -53 | 42 | 3.247033 | 0.011753 |
| Cluster 4<br>*Intraparietal sulcus* | R | 370 | 29 | -44 | 27 | 2.987385 | 0.017403 |
| Cluster 5<br>*Intraparietal sulcus* | L | 2888 | -40 | -59 | 33 | 3.513928 | 0.007916 |
| Cluster 6 | R | 759 | 23 | -2 | 27 | 3.502958 | 0.008044 |

**Supplementary Table 5 – Areas with preferential activation for the *Letter* task (fixed-effects analysis; n=9)**

| | | | *Talairach coordinates* | | | | |
|---|---|---|---|---|---|---|---|
| *Brain areas* | *hemi.* | *size* | *peak x* | *peak y* | *peak z* | *t(6710)* | *p<* |
| Cluster 1 | L | 75 | -19 | -71 | 48 | 4.907614 | 0.000001 |
| Cluster 2 | L | 135 | -16 | -8 | 66 | 5.010720 | 0.000001 |
| *Superior frontal gyrus* | | | | | | | |
| Cluster 3 | L | 2371 | -37 | -2 | 51 | 6.253005 | 0.000000 |
| *Middle frontal gyrus* | | | | | | | |
| Cluster 4 | L | 246 | -37 | -56 | 18 | 5.940580 | 0.000000 |
| Cluster 5 | L | 246 | -36 | 34 | 9 | 5.413554 | 0.000000 |
| *Inferior frontal gyrus* | | | | | | | |
| Cluster 6 | L | 591 | -40 | -47 | -12 | 5.638453 | 0.000000 |
| *Occiptotemporal gyrus* | | | | | | | |

## References


1 Shum, J. *et al.* A brain area for visual numerals. *J Neurosci* **33**, 6709-6715 (2013).

2 Cohen, L. *et al.* The visual word form area: spatial and temporal characterization of an initial stage of reading in normal subjects and posterior split-brain patients. *Brain* **123 ( Pt 2)**, 291-307 (2000).

3 Glezer, L. S. & Riesenhuber, M. Individual variability in location impacts orthographic selectivity in the "visual word form area". *J Neurosci* **33**, 11221-11226 (2013).

4 Cattinelli, I., Borghese, N. A., Gallucci, M. & Paulesu, E. Reading the reading brain: a new meta-analysis of functional imaging data on reading. *Journal of Neurolinguistics* **26**, 214-238 (2013).
